# Vaccinia virus protein K7 is a virulence factor that alters the acute immune response to infection

**DOI:** 10.1099/vir.0.052670-0

**Published:** 2013-07

**Authors:** Camilla T. O. Benfield, Hongwei Ren, Stuart J. Lucas, Basma Bahsoun, Geoffrey L. Smith

**Affiliations:** 1 Department of Pathology, University of Cambridge, Tennis Court Road, Cambridge, CB2 1QP, UK; 2 Department of Virology, Faculty of Medicine, Imperial College London, St. Mary’s Campus, London W2 1PG, UK

## Abstract

*Vaccinia virus* (VACV) encodes many proteins that antagonize the innate immune system including a family of intracellular proteins with a B-cell lymphoma (Bcl)-2-like structure. One of these Bcl-2 proteins called K7 binds Toll-like receptor-adaptor proteins and the DEAD-box RNA helicase DDX3 and thereby inhibits the activation of NF-κB and interferon regulatory factor 3. However, the contribution of K7 to virus virulence is not known. Here a VACV lacking the *K7R* gene (vΔK7) was constructed and compared with control viruses that included a plaque purified wt (vK7), a revertant with the *K7R* gene reinserted (vK7-rev) and a frame-shifted virus in which the translational initiation codon was mutated to prevent K7 protein expression (vK7-fs). Data presented show that loss of K7 does not affect virus replication in cell culture or *in vivo*; however, viruses lacking the K7 protein were less virulent than controls in murine intradermal (i.d.) and intranasal (i.n.) infection models and there was an altered acute immune response to infection. In the i.d. model, vΔK7 induced smaller lesions than controls, and after i.n. infection vΔK7 induced a reduced weight loss and signs of illness, and more rapid clearance of virus from infected tissue. Concomitantly, the intrapulmonary innate immune response to infection with vΔK7 showed increased infiltration of NK cells and CD8^+^ T-cells, enhanced MHC class II expression by macrophages, and enhanced cytolysis of target cells by NK cells and VACV-specific CD8^+^ T-cells. Thus protein K7 is a virulence factor that affects the acute immune response to infection.

## Introduction

*Vaccinia virus* (VACV) is the prototypic member of the genus *Orthopoxvirus* of the family *Poxviridae*. VACV was utilized as the vaccine to eradicate smallpox (Fenner *et al*., 1988), but although VACV induced immunity to *Variola virus*, the cause of smallpox, the vaccine strains used widely in the eradication campaign, such as Lister and New York City Board of Health, caused significant complication rates that are unacceptable in a modern vaccine (Lane *et al.*, 1969). After smallpox eradication, interest in VACV has remained due to its development as an expression vector (Mackett *et al.*, 1982; Panicali & Paoletti, 1982) and the application of engineered VACV strains as tools to study the immune response to virus infection (Bennink *et al.*, 1986; Alcamí & Smith, 1996; Osman *et al.*, 1999) and as live vaccines against infectious diseases (Panicali *et al.*, 1983; Smith *et al.*, 1983a, b) and cancers (Lathe *et al.*, 1987; Walsh & Dolin, 2011). In addition, VACV is an excellent model system for studying virus–host interactions and further study has been prompted by concern about possible bioterrorism with *Variola virus*.**

VACV is a large, complex virus with a dsDNA genome of about 200 kbp that replicates in the cytoplasm (Moss, 2007) and encodes numerous immunomodulatory proteins that antagonize the innate immune response (Smith *et al.*, 1997; Seet *et al.*, 2003). A subgroup of these immunomodulatory proteins have a B-cell lymphoma (Bcl)-2-like structure. For instance, proteins N1 (Aoyagi *et al.*, 2007; Cooray *et al.*, 2007), B14 (Graham *et al.*, 2008), A52 (Graham *et al.*, 2008), K7 (Kalverda *et al.*, 2009) and F1 (Kvansakul *et al.*, 2008) all have had their crystal structures solved and proteins A46, C6, N2 and B22 are also predicted to be members of this family (Graham *et al.*, 2008; González & Esteban, 2010). One of these proteins, K7, is the subject of this study.

VACV strain Western Reserve (WR) protein K7 is a 17.5 kDa intracellular protein that binds to the DEAD-box RNA helicase DDX3, tumour necrosis factor receptor-associated factor 6 (TRAF6) and IL-1 receptor associated kinase 2 (IRAK2) to inhibit activation of interferon regulatory factor (IRF)3 and NF-κB (Schröder *et al.*, 2008). VACV protein A52 also binds TRAF6 and IRAK2 (Harte *et al.*, 2003), which couple engagement of the IL-1 receptor and Toll-like receptors (TLR) to downstream NF-κB signalling. However, deletion of A52 gave an *in vivo* phenotype (Harte *et al.*, 2003) despite the presence of K7, indicating these proteins have non-redundant functions. This may be due to the ability of K7 to bind DDX3, which acts as an adaptor for the TBK1 (TANK binding kinase I)/IKKϵ (IkappaB kinase epsilon) kinases, which mediate IRF activation, and binds directly to the IFN-β promoter (Schröder *et al.*, 2008; Soulat *et al.*, 2008; Mulhern & Bowie, 2010). K7 interacts with a critical diphenylalanine motif at the N terminus of DDX3 (Oda *et al.*, 2009) using a hydrophobic interaction surface that is conserved among VACV Bcl-2-like proteins (Cooray *et al.*, 2007; Graham *et al.*, 2008; Bahar *et al.*, 2011; Benfield *et al.*, 2011; Maluquer de Motes *et al.*, 2011). Thus, K7 can antagonize both TLR-dependent and -independent IFN-β activation (Schröder *et al.*, 2008).

Despite the well-established molecular mechanism of K7, its role *in vivo* is unknown. Here, we have constructed a VACV WR strain lacking K7 (vΔK7) and show that this virus is attenuated in both local and systemic murine infection models. In the absence of K7, virus was cleared more rapidly from the lungs after intranasal (i.n.) infection, and, consistent with this, there were enhanced NK cell- and CD8^+^ T-cell-mediated cytolysis of target cells. Thus, K7 is a virulence factor that affects the acute immune response to infection.

## Results

### K7 expression occurs early after infection and is conserved among orthopoxviruses

**Fig. 1.**
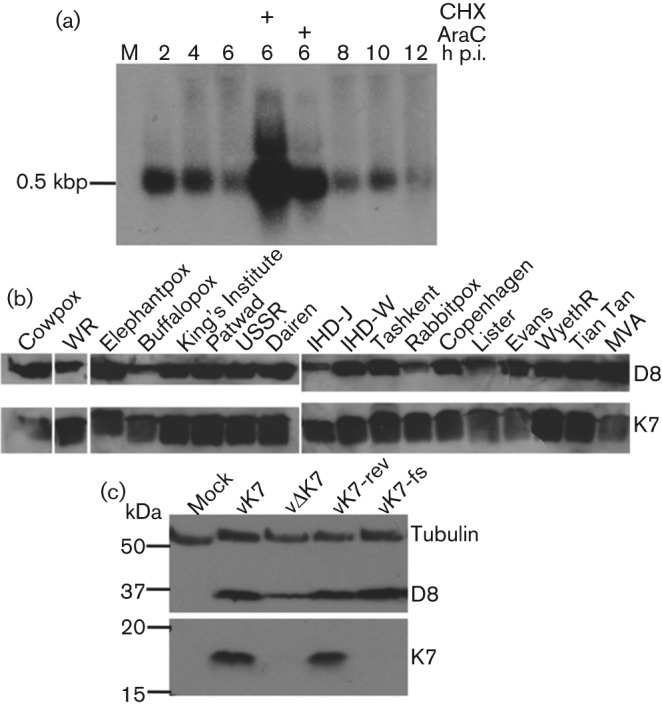
VACV K7 expression occurs early after infection and is conserved among orthopoxviruses. (a) L929 cells were infected with vK7 at 10 p.f.u. per cell or mock-infected (M). Where indicated CHX or AraC was added throughout infection. At the indicated time points total RNA was extracted and 10 µg of RNA was separated by electrophoresis, transferred onto nylon membrane and probed with a ^32^P-labelled K7-specific oligonucleotide. The position of the 0.5 kbp RNA marker is shown. (b) BSC-1 cells were infected with the indicated VACV or CPXV strains for 16 h and cell extracts were immunoblotted using K7-specific antiserum. Virus infection was monitored by immunoblotting to detect VACV D8 protein. WR, VACV strain WR; IHD, International Health Department. (c) BSC-1 cells were infected with the indicated VACVs at 1 p.f.u. per cell overnight or mock-infected. Cell lysates were immunoblotted using anti-alpha-tubulin, anti-K7 and mAb AB1.1 against VACV D8. The positions of molecular size markers are shown in kDa.

The kinetics of K7 transcription from its natural viral promoter were assessed by Northern blotting on total RNA from WR-infected cells using a K7-specific deoxy-oligonucleotide probe (Fig. 1a). This identified a single band of about 500 bp, consistent with the predicted length of the K7 transcript. The steady state K7 mRNA levels were maximal at 2 h post-infection (p.i.) and thereafter declined, but remained detectable until 12 h p.i. The presence of cytosine arabinoside (AraC), an inhibitor of DNA replication, or cycloheximide (CHX), an inhibitor of protein synthesis, enhanced the level of K7 mRNA at 6 h p.i. compared with untreated cells. This is characteristic of early VACV mRNAs, which are transcribed by the virion-associated DNA-dependent RNA polymerase independent of viral protein synthesis or genome replication (Moss, 2007). To test experimentally whether K7 was expressed by different orthopoxviruses, extracts from cells infected with different VACV or cowpox virus (CPXV) strains were immunoblotted with anti-K7 Ab (Fig. 1b, lower panel). K7 was expressed by all 16 VACV strains (including modified VACV Ankara, MVA) and both CPXV strains (Brighton Red and elephantpox virus) tested.

To investigate the contribution of K7 to virus replication and virulence, a VACV deletion mutant lacking the *K7R* gene (vΔK7) was constructed by transient dominant selection (Falkner & Moss, 1990). A plaque purified wt virus (vK7) was isolated from the same intermediate virus. In addition, a revertant virus with the *K7R* gene reinserted into vΔK7 (vK7-rev), and a ‘frame-shifted’ virus in which an additional nucleotide was introduced into the K7 translation initiation codon (vK7-fs) were also constructed as controls. PCR analysis using primers for the *K7R* locus revealed that the *K7R* locus of these viruses was as predicted, and digestion of purified virus genomic DNA separately with *Hin*dIII, *Xho*I and *Nco*I showed that the *Hin*dIII K band was altered in size in vΔK7 as expected, but no other genomic alterations were detectable (Fig. S1, available in JGV Online, and data not shown). Immunoblotting with extracts of cells infected by these viruses confirmed that K7 was expressed by vK7 and vK7-rev but not by vΔK7 or vK7-fs (Fig. 1c). As a control for infection, immunoblotting with mAb AB1.1 directed against the VACV structural protein D8 showed that D8 was expressed by all viruses (Fig. 1c).

**Fig. 2.**
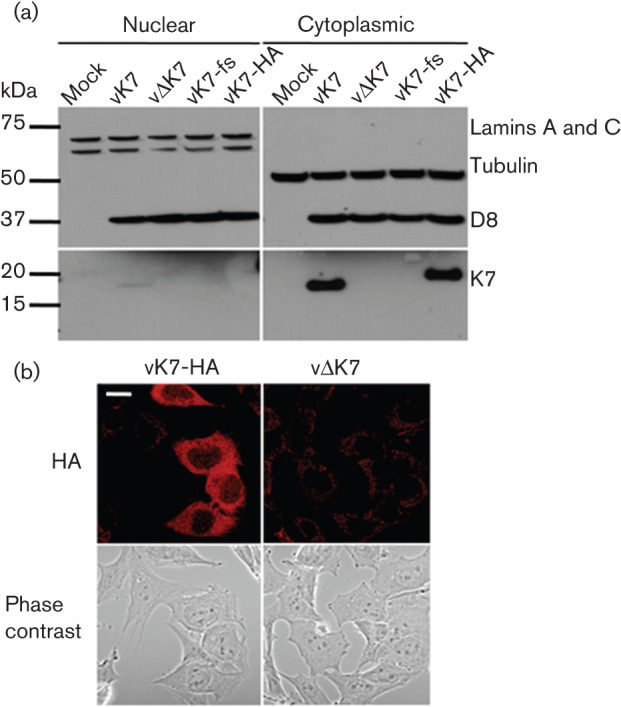
K7 is localized to the cytoplasm. (a) HeLa cells were mock-infected or infected with the indicated viruses at 10 p.f.u. per cell for 16 h. Nuclear and cytoplasmic fractions were then separated and analysed by SDS-PAGE and immunoblotting with the indicated antibodies. A fourfold greater proportion of the nuclear fraction was loaded than for the cytoplasmic fraction. The position of molecular size markers are shown in kDa. (b) HeLa cells were infected with vK7-HA or vΔK7 at 5 p.f.u. per cell for 5 h. After fixation and permeabilization, cells were stained with mouse anti-HA followed by anti-mouse Alexa Fluor 546 (red)-conjugated secondary antibody and viewed by confocal microscopy. Bar, 20 µm.

The subcellular localization of K7 was assessed using biochemical fractionation of infected HeLa cells followed by anti-K7 immunoblotting (Fig. 2a). K7 was detected within the cytoplasmic fraction of cells infected with either wt virus or vK7-HA, which expresses N-terminally haemaggltinin (HA) epitope-tagged K7 from its natural locus (Methods). Immunofluorescence on fixed cells confirmed the cytoplasmic distribution of HA in vK7-HA-infected cells, compared with only background staining in cells infected with vΔK7 (Fig. 2b).

### K7 is non-essential for replication but contributes to VACV virulence

**Fig. 3.**
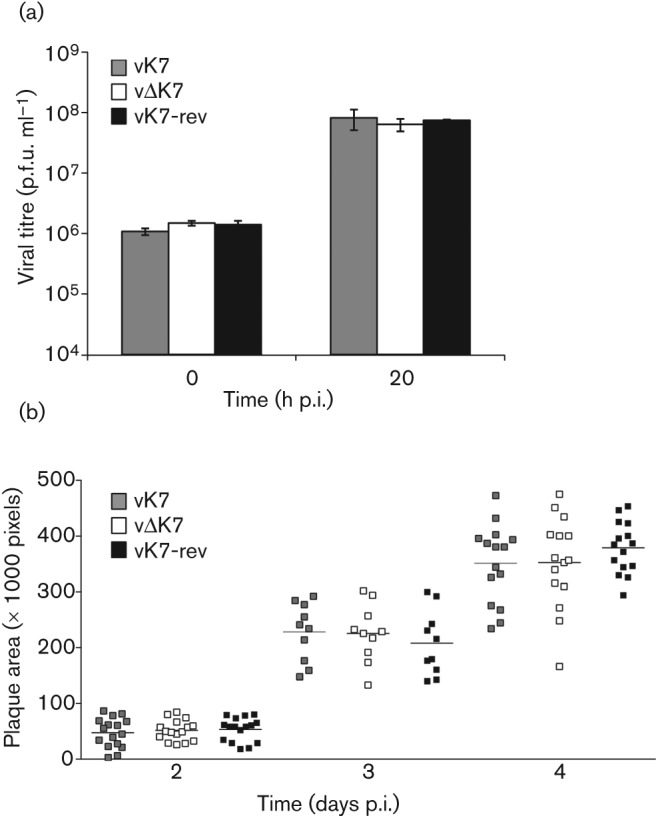
K7 is non-essential for VACV replication and spread in cell culture. (a) BSC-1 cells were infected with indicated viruses at 10 p.f.u. per cell and were harvested at the indicated times. Cells were disrupted by freeze–thawing and sonication and infectious virus was titrated by plaque assay. Data shown are mean±sd of 2 separate experiments. (b) Scatter plots with means (horizontal bars) of plaque sizes of vK7, vΔK7 and vK7-rev in BSC-1 cells infected with 50–100 p.f.u. per well and incubated for 2, 3 or 4 days. Plaque sizes (*n* = 15) were measured using ImagePro 4.0 analysis software.

The replication of vΔK7 in cell culture was measured after infection at high m.o.i. and found to be indistinguishable from controls (Fig. 3a). Similarly, the plaque size of vΔK7 on BSC-1 cells was indistinguishable from control viruses, indicating that K7 was not needed for normal cell-to-cell spread (Fig. 3b). Hence K7 is non-essential for VACV replication in cell culture.

**Fig. 4.**
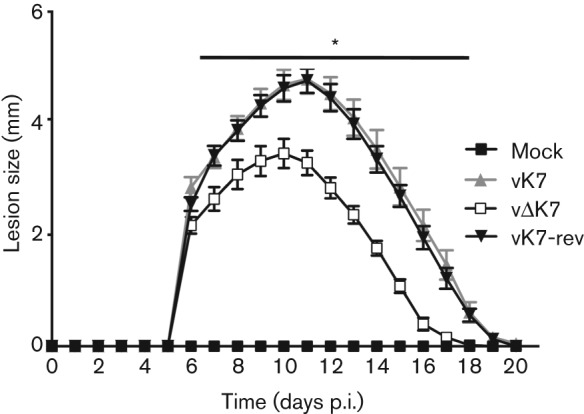
K7 is a virulence factor in the i.d. infection model. C57BL/6 mice (*n* = 5 per group) were mock-infected or infected i.d. with 1×10^4^ p.f.u. per ear of the indicated viruses into both ears. Lesion sizes were measured daily and data are expressed as mean±sem. Infection with vΔK7 induced a smaller lesion compared with vK7 and vK7-rev (*, *P*<0.05).

**Fig. 5.**
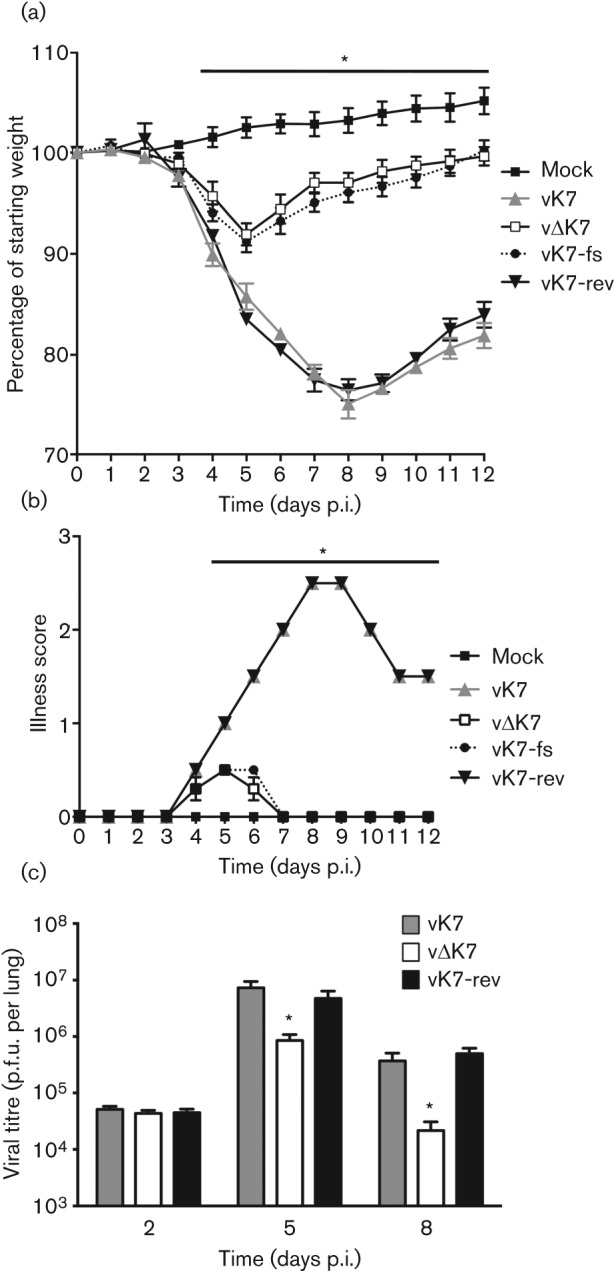
Deletion of *K7R* causes attenuation in the i.n. infection model and more rapid viral clearance. (a, b) BALB/c mice (*n* = 5 per group) were mock-infected or infected i.n. with 7×10^3^ p.f.u. per mouse and body weights (a) or signs of illness (described by Alcamí & Smith, 1992) (b) were measured daily. Body weight is expressed as the percentage of the mean weight of the same group of animals on day 0. (c) BALB/c mice (*n* = 5 per group) were mock-infected or infected i.n. with 1×10^4^ p.f.u. per mouse. On the days indicated, infectious virus in lung cell extracts was measured by plaque assay. The Data shown are mean±sem. *, Significant difference between vΔK7 and other viruses (*P*<0.05).

To assess whether K7 contributed to VACV virulence in the intradermal (i.d.) murine model of infection (Tscharke & Smith, 1999), vΔK7 or control viruses were inoculated i.d. in the ear pinna of C57BL/6 mice and the size of the resulting lesions was measured daily (Fig. 4). Infection with vΔK7 induced a smaller lesion compared with vK7 and vK7-rev, and these differences were statistically significant (two-tailed Student’s *t*-test, *P*<0.05) over days 7–18. Next the virulence of vΔK7 was compared with that of control viruses by i.n. infection of mice (Williamson *et al.*, 1990). vΔK7 induced significantly less weight loss (Fig. 5a) and fewer signs of illness (Alcamí & Smith, 1992) (Fig. 5b) than the K7-expressing viruses vK7 and vK7-rev. An additional control virus, vK7-fs, containing an extra nucleotide to disrupt the translational start codon of the K7 ORF, behaved equivalently to vΔK7 (Fig. 5a, b). This confirmed that the K7 protein was responsible for the attenuated phenotype. Using a slightly lower virus dose (5×10^3^ p.f.u.), vK7 and vK7-rev viruses caused 25 % reduction in body weight, while vΔK7 caused no weight loss or signs of illness (data not shown). This attenuation correlated with significantly lower viral titre in the infected lungs of vΔK7-infected mice compared with controls at days 5 and 8 p.i. (Fig. 5c). At day 2 p.i. all virus titres were increased above that of the inoculation dose but were equivalent between all groups showing that K7 is not needed for replication *in vivo*. This confirmed that subsequent differences were due to more efficient clearance of vΔK7 rather than replicative differences and suggested that vΔK7 might induce a more robust antiviral immune response. This was investigated by assessing immune cell infiltration into infected lungs.

### K7 alters leukocyte recruitment following i.n. VACV infection

**Fig. 6.**
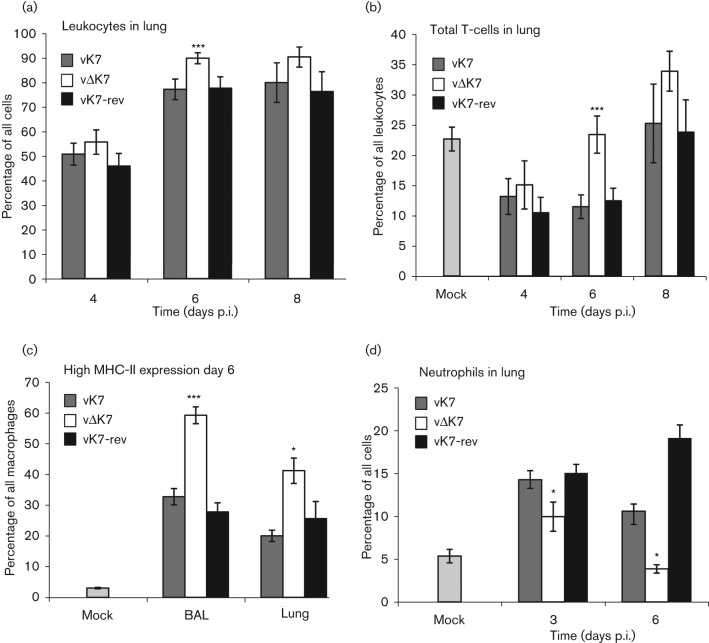
K7 affects immune cell recruitment into the lung. Mice (*n* = 5 per group) were mock-infected or infected i.n. (a–d) with 1×10^4^ p.f.u. On the days indicated, cells from lungs were washed, counted and stained for flow cytometry. Percentage of (a) leukocytes (as a proportion of all cells recovered), (b) total T-cells (as a proportion of all leukocytes), (c) surface expression of MHC class II (as a proportion of all macrophages) and (d) neutrophils (as a proportion of all cells recovered) in the lung. A forward/side scatter gate was applied to exclude cell debris and larger stromal cells from analysis. T-cells were defined as small, non-granular CD3^+^ cells. Macrophages were defined as large, granular F4/80^+^ and CD11b^+^ cells. Granulocytes were defined as granular Ly6G^+^ cells. Data are presented as mean±sd. Statistical analyses were by one-factor ANOVA with Bonferroni post-tests for pairwise comparisons; asterisks indicate significant difference between vΔK7 and other viruses: *, *P*<0.05; ***, *P*<0.001. BAL, bronchoalveolar lavage.

The kinetics and composition of leukocyte recruitment into the lung following i.n. inoculation of VACV WR was reported previously (Reading & Smith, 2003a). To determine the impact of K7 on leukocyte recruitment, infiltrating cells were analysed by flow cytometry at several times p.i. with vΔK7, vK7 or vK7-rev (Fig. 6). In mice infected with vΔK7 there was no statistically significant change in the overall number of infiltrating cells harvested from lung tissue compared to the controls, although there appeared to be a slight increase at day 6 (data not shown). However, a higher proportion of these cells expressed the common leukocyte antigen CD45 on day 6 compared with the controls (Fig. 6a). At this time there was a substantial increase in infiltrating T-cells suggesting that the overall increase was dominated by T-cells (Fig. 6b). Furthermore, after vΔK7 infection there were approximately twice as many activated macrophages in both the alveolar space (bronchoalveolar lavage, BAL) and the lung tissue on day 6 compared with controls, as shown by high surface expression of MHC class II (Fig. 6c). Concomitantly, there were fewer neutrophils (as a proportion of total cells recovered) in the lung tissue of vΔK7-infected mice relative to controls at days 3 and 6 p.i. (Fig. 6d).

### vΔK7 induces enhanced intrapulmonary NK- and CD8^+^ T-cell-dependent cytotoxicity

**Fig. 7.**
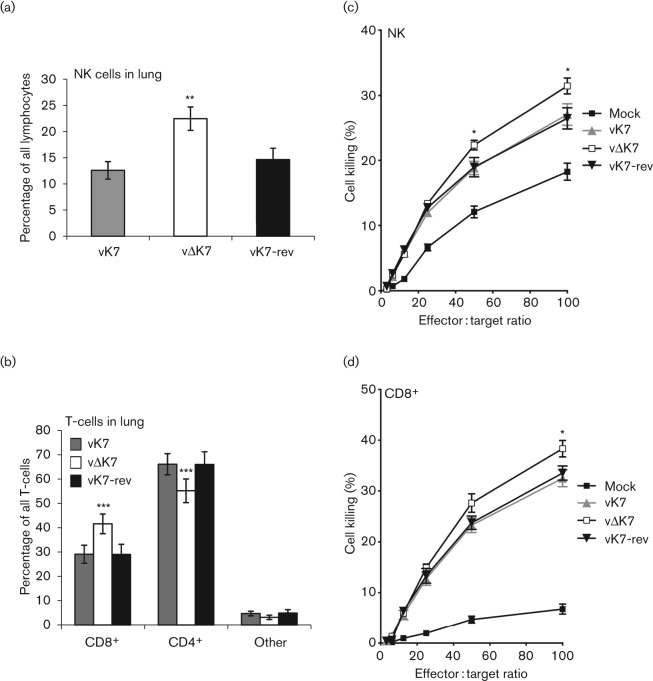
vΔK7 induces augmented intrapulmonary cytolysis by NK and CD8^+^ T-cells. BALB/c mice (*n* = 5 per group) were infected i.n. with the indicated viruses at 7×10^3^ p.f.u. per mouse and lung tissue was harvested on day 6. Flow cytometry was used to define the percentage of NK cells (CD3^−^, DX5^+^; expressed as a proportion of all lymphocytes) (a) and T-cell subsets CD4^+^ and CD8^+^ (expressed as a proportion of all T-cells) (b). Chromium-release cytotoxicity assays were performed using total lung cell suspensions as effector cells. Yac-1 cells were used as NK cell targets (c) and VACV-infected P815 cells were used as CD8^+^ T-cell targets (d). Data are presented as mean±sd for cytometry (a, b) and mean±sem for cytotoxicity assays (c, d). Asterisks indicate significant difference between vΔK7 and other viruses: *, *P*<0.05; **, *P*<0.01; ***, *P*<0.001.

Since NK and CD8^+^ cytotoxic T-lymphocytes (CTL) play crucial roles in controlling poxvirus infections *in vivo* (Bukowski *et al.*, 1983; Xu *et al.*, 2004; Parker *et al.*, 2007; Martinez *et al.*, 2010), these cell populations were analysed in the lung 6 days after i.n. infection. There were significantly more NK cells in the lungs of vΔK7-infected mice, both as a proportion of total lymphocytes (Fig. 7a) and in absolute numbers (data not shown). In addition, a significantly higher proportion of infiltrating T-cells were CD8^+^ CTL, rather than CD4^+^ cells, following vΔK7 infection compared with vK7 or vK7-rev (Fig. 7b). To assess the functional consequence of the enhanced recruitment of NK cells, NK-sensitive Yac-1 target cells were labelled with ^51^Cr and incubated with single-cell suspensions of homogenized lungs. A significantly higher level of NK-mediated target lysis was seen (for effector : target ratios of 50 and 100; Student’s *t*-test, *P*<0.05) for mice infected with vΔK7 compared with control viruses (Fig. 7c). Similarly, enhanced killing of ^51^Cr-loaded VACV-infected P815 target cells by CD8^+^ CTL was observed for vΔK7 at an effector : target ratio of 100 (Fig. 7d). These data demonstrate that removal of K7 from VACV increases the overall pulmonary NK- and CTL-dependent cytotoxic responses after infection.

## Discussion

This report provides characterization of the contribution of VACV WR protein K7 to virus replication, spread and virulence and demonstrates that K7 is a virulence factor that affects the acute immune response to infection. A virus lacking K7 replicated normally in cell culture (Fig. 3) and *in vivo* (Fig. 5c d 2), but was less virulent than control viruses in both i.d. (Fig. 4) and i.n. (Fig. 5a, b) infection models and induced stronger pulmonary NK and CD8^+^ T-cell responses (Fig. 7). Consistent with this, infectious virus was cleared from lungs more rapidly after infection with vΔK7 than control viruses (Fig. 5c).

K7 is a member of the Bcl-2 family of VACV proteins (Graham *et al.*, 2008; Kalverde *et al.*, 2009; González & Esteban, 2010) and shares several features with other proteins of this family. These proteins are characterized by inhibiting either innate immune signalling pathways (N1, B14, A52, A46, C6) and/or apoptosis (N1 and F1), and N1 (Kotwal *et al.*, 1989; Bartlett *et al.*, 2002; Cooray *et al.*, 2007; Maluquer de Motes *et al.*, 2011), B14 (Chen *et al.*, 2006, 2008), A52 (Harte *et al.*, 2003), A46 (Stack *et al.*, 2005), C6 (García-Arriaza *et al.*, 2011; Unterholzner *et al.*, 2011; Sumner *et al.*, 2013) and K7 (this report) are all non-essential for virus replication but mutant viruses lacking each protein separately all show attenuated phenotypes *in vivo*. Therefore, these proteins have non-redundant functions.

Characterization of *K7R* gene transcription by Northern blotting showed a single 500 bp mRNA whose expression was maximal at 2 h p.i. (Fig. 1a), consistent with the transcription start site and immediate-early kinetics assigned to *K7R* in genome-wide expression studies (Assarsson *et al.*, 2008; Yang *et al.*, 2010). The K7 protein was predominantly cytoplasmic during virus infection (Fig. 2a, b), in contrast to the nuclear and cytoplasmic distribution seen following plasmid transfection (Schröder *et al.*, 2008). K7 shows a high level of conservation between orthopoxviruses [Fig. 1b and Schröder *et al.* (2008)] and has a defined molecular mechanism (Schröder *et al.*, 2008; Kalverda *et al.*, 2009; Oda *et al.*, 2009), making it attractive for further study. This report shows that deletion of a single cytoplasmic protein that blocks NF-κB activation and IFN-β induction can alter both virus virulence and the primary immune response to infection, promoting a Th1 (type 1 T-helper cell)-skewed response, despite the presence of other VACV inhibitors of NF-κB and IRF3.

The attenuation of vΔK7 *in vivo* was not due to a defect in virus replication because both virus yield and plaque size in cell culture (Fig. 3), and replication shortly after infection *in vivo* (Fig. 5c) were equivalent with control viruses. The two murine infection models used here are distinct and complementary for assessing VACV virulence. The i.d. infection of ear pinnae mimics dermal vaccination: only a localized lesion develops and immunity against VACV challenge is induced (Tscharke & Smith, 1999; Tscharke *et al.*, 2002; Jacobs *et al.*, 2006). On the other hand, i.n. infection leads to acute pneumonitis followed by dissemination to secondary sites of infection (including brain, spleen and liver) and systemic signs of illness (Williamson *et al.*, 1990; Alcamí & Smith, 1992; Reading & Smith, 2003a). VACV inoculation by the two different routes induces different inflammatory responses (Reading & Smith, 2003a), and approximately half of the single gene mutants which have been tested in both models exhibit a phenotype in only one model (Tscharke *et al.*, 2002). Moreover, deletion of VACV immunomodulators does not lead to predictable phenotypic consequences, and there are examples where virulence was enhanced rather than reduced (Alcamí & Smith, 1992, 1996; Reading *et al.*, 2003b; Clark *et al.*, 2006). Notably, deletion of K7 results in attenuation of WR in both infection models.

The immune response to infection by vΔK7 was analysed by flow cytometry and this revealed that different cell populations were recruited to the infected tissue (Figs. 6 and 7). Infection (i.n.) with vΔK7 led to significant increases in total T-cell influx, in CD8^+^ T-cells (expressed as a percentage of total T-cells), in NK cell recruitment and in MHC class II expression by macrophages in the lung parenchyma or bronchoalveolar space (relative to control infections). Together, these alterations indicate that loss of K7 induced a more robust Th1-cell-mediated immune response. The most marked differences in cellular composition were seen at day 6 p.i. (Fig. 6). At this time, the net cytotoxic activity of pulmonary NK and CD8^+^ T-cells was significantly increased (Fig. 7), and viral titres in the lungs of vΔK7-infected mice were significantly reduced from 5 days p.i. compared with control virus infections (Fig. 5c). These data are consistent with a recent report showing that CD8^+^ T-cells (not CD4^+^ T-cells or antibodies) are necessary and sufficient for recovery from primary WR infection in the murine i.n. model used here (Goulding *et al.*, 2012).

The targeted deletion of immunomodulators from VACV has the potential to improve the utility of VACV as a vaccine by enhancing safety and/or immunogenicity. For instance, with VACV proteins that are secreted from the infected cell, it was shown that deletion of VACV gene *A41L* encoding a secreted CC chemokine binding protein (Ng *et al.*, 2001; Bahar *et al.*, 2008) enhanced the primary CD8^+^ T-cell responses to infection although virulence was enhanced slightly (Clark *et al.*, 2006). Removal of a secreted IL-1β binding protein, which is encoded by several VACV strains including MVA (Alcamí & Smith, 1992; Spriggs *et al.*, 1992; Blanchard *et al.*, 1998), also created a more immunogenic MVA strain (Staib *et al.*, 2005; Cottingham *et al.*, 2008). The immunogenicity of VACV has also been improved by removal of genes encoding other secreted proteins that bind IL-18 (Smith *et al.*, 2000; Reading & Smith, 2003b; Falivene *et al.*, 2012) or type I IFN (Colamonici *et al.*, 1995; Symons *et al.*, 1995; Gómez *et al.*, 2012) or type II IFN (Alcamí & Smith, 1995; Mossman *et al.*, 1995; Gómez *et al.*, 2012).

Similarly, VACV intracellular proteins also modulate the inflammatory response. For instance, deletion of the steroid biosynthetic enzyme 3β-hydroxysteroid dehydrogenase enhanced IFN-γ production and VACV-specific CD8^+^ CTL (Moore & Smith, 1992; Reading *et al.*, 2003a). In addition, deletion of N1 (Jacobs *et al.*, 2008), B14 (Chen *et al.*, 2006) and C16 (Fahy *et al.*, 2008) also altered the inflammatory response to infection. This study demonstrates that removal of the intracellular protein K7, which has a known structure and mechanism of action, simultaneously reduced the virulence and enhanced the immunogenicity of VACV despite the presence of other NF-κB, IRF3 and IFN antagonists. Both these phenotypic changes are desirable for VACV-based vaccines. While VACV WR provides a well-characterized and virulent virus strain in which to test phenotypes, it is not a vaccine strain. MVA is a promising candidate for VACV-vectored vaccines (Sutter & Staib, 2003) and although it lacks many immunomodulators (Antoine *et al.*, 1998; Blanchard *et al.*, 1998), it expresses an identical version of K7 to that studied here [Fig. 1b and Schröder *et al.* (2008)]. Our report indicates that deletion of K7 is a promising strategy for enhancing the immunogenicity and utility of VACV as a vaccine.

## Methods

### 

#### Cell culture.

BSC-1 and L929 cells were grown in Dulbecco’s modified Eagle’s medium (DMEM; Gibco) supplemented with 10 % FBS (Harlan Seralab) and penicillin/streptomycin (50 µg ml^−1^; Gibco). HeLa cells were maintained in modified Eagle’s medium (Gibco) containing 10 % FBS, non-essential amino acids (Sigma M7145) and antibiotics as above. Murine tumour cells YAC-1 (lymphoma), P815 (mastocytoma) and EL4 (thymoma) were grown in RPMI 1640 (Gibco) containing 10 % FBS and antibiotics as above. All cells were cultured at 37 °C in a humidified 5 % CO_2_ atmosphere.

#### Antibodies.

Anti-K7 polyclonal antibody (Schröder *et al.*, 2008) and the mouse mAb AB1.1 against VACV protein D8 (Parkinson & Smith, 1994) were described previously. Mouse anti-lamins A and C (Abcam ab8984), mouse anti-tubulin (Millipore 05-829) and mouse anti-HA (Covance MMS-101P) were also used.

#### Construction of recombinant VACVs.

The following oligonucleotide primers were used for PCR: 039U (5′-AACTTCTAGATTCACCATTACTTCTTCCATGTCC-3′); 039D (5′-TGATGAATTCGGGGTTGGGTGTAAGATTGG-3′); 039N (5′-*CCCCTATATCAGACTATCTCAC*AAAAGACAGTAGC-3′); and 039C (5′-*GTGAGATAGTCTGATATAGGGG*TCTTCATAACGC-3′).

Using 039U and 039D, the *K7R* ORF with 339 bp of upstream and 323 bp of downstream flanking sequence was amplified from VACV WR genomic DNA by PCR. The flanking sequences alone were amplified using 039U and 039N and 039C and 039D, respectively, and then spliced together via the overlapping sequences of 039N and 039C (italicized) in a second amplification with 039U and 039D. PCR products were cloned into the *Eco*RI site of plasmid pSJH7 (Hughes *et al.*, 1991) to produce pSJH7-K7 and pSJH7-ΔK7. The plasmid pSJH7-K7-fs was constructed by site-directed mutagenesis using pSJH7-K7 as the template and contained an extra adenine within the translation initiation codon (i.e. ATAG). The plasmid pSJH7-K7-HA was constructed with an N-terminal HA tag in-frame with the K7R ORF. All plasmids were sequenced to ensure their fidelity. CV-1 cells were infected with VACV strain WR and transfected with pSJH7-ΔK7 and vΔK7 was isolated by transient dominant selection as described previously (Falkner & Moss, 1990), along with a wt isolate (vK7) derived from the same intermediate virus. Similarly, vΔK7-infected cells were transfected with pSJH7-K7 to create a revertant virus, vK7-rev, with pSJH7-K7-fs to create vK7-fs, or with pSJH7-K7-HA to create the vK7-HA virus. Virus infectivity and plaque morphology were assessed by plaque titration on BSC-1 cells.

#### Northern blotting.

L929 cells were infected with VACV WR at 10 p.f.u. per cell and, at various times p.i., total RNA was collected using Trizol. Cycloheximide (CHX; Sigma Aldrich, UK), at 100 µg ml^−1^, or cytosine arabinoside (AraC; Sigma Aldrich, UK), at 40 µg ml^−1^, was added where indicated during the 1 h adsorption period and for 6 h thereafter. Total RNA (10 µg) for each sample was denatured by glyoxylation immediately prior to electrophoresis in a 1.4 % agarose gel and 0.5 M sodium phosphate (pH 7) running buffer. RNA was transferred by capillary transfer onto Hybond-N^+^ membrane, UV cross-linked to the membrane and then de-glyoxylated using boiling 20 mM Tris/HCl pH 8. The blot was then pre-hybridized in ULTRAhyb-Oligo Hybridization buffer (Ambion) for 30 min at 42 °C before 10^6^ counts min^−1^ ml^−1^ of ^32^P-labelled deoxy-oligonucleotide probe (5′-GTGGTCTCCTTCGCTCATAGCTTCGACAATCTC-3′) was added and hybridized overnight at 42 °C to detect K7R mRNA. The probe was prepared by 5′-end labelling 10 pmol of oligonucleotide with 20 pmol γ-^32^P ATP using T4 polynucleotide kinase (NEB). Following hybridization, the membrane was washed in 2×SSC (saline-sodium citrate buffer, 0.3M sodium chloride, 0.03M sodium citrate)/0.5 % SDS twice for 30 min each at 42 °C before exposing it to X-ray film. To allow sizing, RNA markers were run on the gel, stained with ethidium bromide and then aligned with the membrane.

#### Confocal microscopy.

HeLa cells were grown on glass coverslips, fixed using 4 % paraformaldehyde (Electron Microscope Science, Germany) in 250 mM HEPES/PBS and permeabilized using 0.1 % Triton X-100 (Sigma Aldrich). The cells were then pre-incubated in blocking buffer (10 % FBS in PBS) for 1 h, incubated with primary antibody for 1 h and then incubated with anti-mouse Alexa Fluor 546-conjugated secondary antibodies (Invitrogen) for 30 min. Coverslips were mounted in DAPI and examined with a Zeiss LSM5 Pascal scanning confocal microscope using Zeiss LSM software.

#### Cellular fractionation.

Cells were washed twice with ice-cold lysis buffer (20 mM HEPES pH 7.8, 0.5 mM DTT, 0.5 mM MgCl_2_ supplemented with Roche protease inhibitor tablets) prior to separation of nuclear and cytoplasmic fractions as previously described (Unterholzner *et al.*, 2011).

#### Immunoblotting.

Cell lysates were prepared and proteins detected as previously described (Bartlett *et al.*, 2002).

#### Virus growth curves.

BSC-1 cells were infected with each virus in duplicate at 10 p.f.u. per cell, and single cycle virus growth was determined as previously described (Chen *et al.*, 2006).

#### Plaque size assay.

Plaque size was measured microscopically on BSC-1 monolayers as previously described (Doceul *et al.*, 2010).

#### Murine infection models.

For i.d. inoculations, female, 6–8 week old C57BL/6 mice were infected as described previously (Tscharke & Smith, 1999; Tscharke *et al.*, 2002). For i.n. infection, female, 6–8 week old BALB/c mice were inoculated under anaesthetic with the indicated virus dose in 20 µl of PBS supplemented with 0.1 % BSA (or mock-infected with the same diluent), and monitored as described previously (Williamson *et al.*, 1990; Alcamí & Smith, 1992). Inocula were routinely titrated by plaque assay to confirm the dose administered. Experiments were conducted under the appropriate licence and regulations stipulated by the Animals (Scientific Procedures) Act 1986, UK Government. All *in vivo* data shown are from one representative experiment, and all experiments were performed at least twice.

#### Determination of lung viral titres.

The lungs were homogenized and washed through a 70 µm nylon mesh using DMEM and10 % FBS. Cells were then frozen and thawed three times, and sonicated thoroughly to liberate intracellular virus. Infectious virus was titrated in duplicate by plaque assay on BSC-1 cell monolayers.

#### Cell preparation and staining for flow cytometry.

Bronchoalveolar lavage (BAL) fluids were obtained, and lung cells were prepared as described previously (Reading & Smith, 2003a; Chen *et al.*, 2006; Clark *et al.*, 2006). Fluorophore-antibody conjugates (used at recommended dilutions) were: allophycocyanin (APC)-rat anti-mouse CD45, FITC-anti-mouse CD3, APC-rat anti-mouse CD4 (BD), phycoerythrin (PE)-rat anti-mouse CD8a (BD), PE-rat anti-mouse I-A/I-E (MHC II) (BD), FITC-rat anti-mouse CD11b (Serotec), PE-rat anti-mouse Ly6G (BD) and PE-Cy5.5-rat anti-mouse F4/80 (Caltag). Stained cells were washed, fixed using 1 % paraformaldehyde in PBS and analysed on a BD FacsCalibur.

#### Chromium-release cytotoxicity assay.

NK cell cytotoxicity and VACV-specific CTL activity was assayed with a standard ^51^Cr-release assay using the previously described protocol (Clark *et al.*, 2006). NK-mediated lysis was tested on uninfected YAC-1 cells, while VACV-infected P815 cells (H-2d, mastocytoma) were used as targets for VACV-specific CTL lysis. The percentage of specific ^51^Cr release was calculated as specific lysis  =  [(experimental release−spontaneous release)/(total detergent release−spontaneous release)]×100. The spontaneous release values were always <10 % of total lysis.

#### Statistical analysis.

Unless otherwise stated, data were analysed using a two-tailed Student’s *t*-test. Unless otherwise stated, datasets were compared with the vΔK7-infected samples. The threshold for significance was *P*<0.05, and is indicated by *, *P*<0.05; **, *P*<0.01; ***, *P*<0.001.
